# Voltammetric determination of itopride using carbon paste electrode modified with Gd doped TiO2 nanotubes

**DOI:** 10.3906/kim-2003-56

**Published:** 2020-08-18

**Authors:** Abdulaziz Nabil AMRO, Khadijah EMRAN, Hessah ALANAZI

**Affiliations:** 1 Department of Chemistry, College of Science, Taibah University, Madinah Saudi Arabia; 2 Department of Chemistry, College of Science and Art, Al Jouf University, Qurayyat Saudi Arabia

**Keywords:** Gd-TiO_2_ nanotube, hydrothermal, cyclic voltammetry, itopride, nanocomposite electrode

## Abstract

In the present work TiO_2_ nanotubes (TNT) have been synthesized by alkaline hydrothermal transformation. Then they have been doped with Gd element. Characterizations of doped and undoped TNT have been done with TEM and SEM. The chemical composition was analyzed by EDX, Raman and FTIR spectroscopy. The crystal structure was characterized by XRD. Carbon paste electrode has been fabricated and mixed with Gd doped and undoped TNT to form a nanocomposite working electrode. Comparison of bare carbon paste electrode and Gd doped and undoped TNT carbon paste electrode for 1.0 ×10^−3^ M K_4_ [Fe(CN)_6_] voltammetric analysis; it was observed that Gd doped TNT modified electrode has advantage of high sensitivity. Gd doped TNT modified electrode has been used as working electrode for itopride assay in a pharmaceutical formulation. Cyclic voltammetry analysis showed high correlation coefficient of 0.9973 for itopride (0.04–0.2 mg/mL) with a limit of detection (LOD) and limit of quantitation values (LOQ) of 2.9 and 23.0 μg.mL^−1^ respectively.

## 1. Introduction

**Figure 1:**
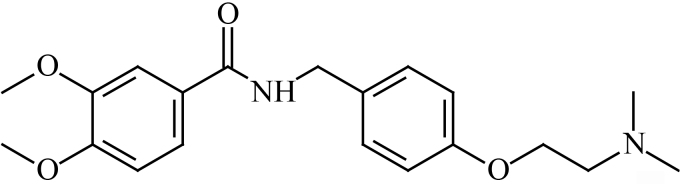
Chemical structure of itopride hydrochloride.

Itopride hydrochloride IUPAC name: (N-[4-[2-(dimethyl amino)-ethoxy] benzyl]-3, 4 dimethoxy benzamide hydrochloride) as a benzamide derivative compound is shown in Figure 1. Itopride acts as inhibitor to acetylcholine esterase enzyme, dopamine, and a gastrokinetic effect [1,2]. This pharmaceutical compound is effective for the gastrointestinal symptoms in addition to functional dyspepsia and chronic gastritis [3].

Most of the studies have used high performance liquid chromatography (HPLC) for itopride assay in pharmaceutical formulation [3–11]. Spectrophotometric methods have also been used for itopride quantitation [12–14]. Pure and combined dosage forms have been analyzed using Potentiometric method [15]. Ru(bpy)^2+^_3_ doped silica nanoparticles/chitosan composite films modified electrode has been used as Electrogenerated Chemiluminescence sensor for itopride assay [16]. In recent work Voltammetry has been applied for itopride assay using commercially available platinum electrode [17].

Electrochemical analysis methods have grown in recent years as alternative to other analytical methods [18–20]. Electroanalytical methods have some advantages making them better alternative such as high sensitivity, selectivity, low instrumentation and running cost, easy to handle and short analysis time. Several parameters have played a role in the performance of the electroanalytical methods; one of them is working electrode. Several studies have been done to enhance the performance of working electrodes. In this field carbon paste working electrode achieved a special importance; this importance comes from its simple fabrication steps, in addition to low cost and wide potential window. Various materials such as nanoparticles could be added to carbon paste mixture during preparation to enhance the sensitivity and selectivity of the electrodes [21,22].

Titanium dioxide (TiO_2_) is belonging to metal oxide semiconductor that considered as the perfect materials in widespread environmental and medical applications [23–26]. TiO_2_-based nanomaterials as nanotubes have been intensively studied and widely used due to their excellent electrolytic and electrolysis performance, high chemical stability and efficiency, nontoxicity and low cost [27–30]. The high cation exchange capacity on titanium dioxide nanotubes (TNT) provides the possibility of achieving a high loading of active compound on it, which makes it one on the best sensor. Otherwise, the high specific surface area and absence of micropores in TNT which facilitate transport of reagent the active sites. The bandwidth between the valence and conduction bands limits its activity [31]. By addressing this issue, doping with elements as rare-earth that have a large atomic number have been devoted [32–35]. The electronic energy levels in rare-earth elements are rich and improve its photocatalytic and electrocatalytic activity.

In this study, TiO_2_ nanotubes have been prepared then doped with Gd, after the characterization Gd doped TiO_2_ nanotubes. Carbon paste electrode has been mixed with different doses of TiO_2_ nanotubes and Gd doped TiO_2_ nanotubes, cyclic voltammetry has been established to study the performance of each fabricated electrode, then the electrode exhibited the best performance that has been used in voltammetric analysis of itopride in a pharmaceutical formulation.

## 2. Experimental

### 2.1. Materials and reagents

The standard pharmaceutical formulation of itopride hydrochloride was obtained from Trium pharma (Jordan), sodium sulphate anhydrous Na_2_SO_4_ from Janssen Chemica. The supporting electrolyte 1.0 M Na_2_SO_4_ was prepared using Milli-Q water. 1.0 M Na_2_SO_4_ supporting electrolyte was used for the preparation of stock solutions and standard working solutions. K_4_Fe(CN)_6_.3H_2_O was obtained from (Sigma Aldrich), Graphit powder from (BDH), and Paraffin liquid light BP from (Pacegrove).

### 2.2. Synthesis of TNTs

The method of preparation of TNT and doped Gd-TNT was based on alkaline hydrothermal transformation. A weighted amount of TiO_2_ powder [P25, (99.5%, 21 nm), Sigma-Aldrich, USA] was added to 30 mL of 10 mol dm^−3^ potassium hydroxide [KOH,Sigma-Aldrich, USA] solution. After stirring for 30 min and the mixture was transferred into a Teflon-lined stainless-steel autoclave and was heated for 24 h at 150 °C. The white Powderly precipitate was thoroughly washed with deionized water then with dilute HCl until the pH of washing solution reached 6.5 then with deionized water again, followed by drying for 10 h at 90 °C, and calcinating at 400 °C for 2 h. Gd-TNT was synthesized by adding Gd(NO_3_)_3_ to the TiO_2_ in the KOH solution followed by the hydrothermal and postsynthetic treatments as described above for the undoped TNTs [35].

### 2.3. Characterizations

The morphology of undoped TNTs and doped Gd-TNTs was examined by Transmission Electron Microscope (TEM, JEOL JEM 1400, Japan) and scanning electron microscopy equipped (SEM, Superscan SS-550, Shimadzu, Japan). The chemical composition was analyzed by energy dispersive X-ray analysis equipped (EDX, Superscan SS-550, Shimadzu, Japan). The crystal structure of the as-prepared sensors were characterized by a X-ray diffraction (XRD, Shimadzu, XRD-7000, Japan) at 40 kV and 30 mA, using CuKα incident beam (λ = 0.154nm). Raman spectroscopy was performed on a Raman microscope (Raman, Sentrarra, Bruker, USA) from 50 cm^−1^ to 1200 cm^−1^. Infrared (FT-IR) absorption spectra of the KCl disks containing powder samples were recorded on a Thermo IS-10 instrument FT-IR spectrometer (Thermo Fisher Scientific Inc., Madison, WI, USA) at a resolution of 4 cm^−1^ in the range of 400–4000 cm^−1^.

### 2.4. Modified carbon paste electrode fabrication

For fabrication of carbon paste modified electrodes, graphite powder, TNT, and Gd-TNT have been mixed as in the Table 1.

**Table 1:** Quantities of fabricated electrodes contents.

Electrode code	Graphite powder (mg)	TNT(mg)	Gd-TNT(mg)
C paste	200	0	0
F1	200	25	0
F2	200	50	0
G1	200	0	25
G2	200	0	50

After that mixture powder was dispersed in 1.0 mL dimethyl formamide (DMF) then homogenized for 20 min in ultrasonic bath. After that DMF was vaporized from the mixture using oven at 80 °C overnight. Dry mixture was mixed with 100 μL paraffin oil using spatula. Micropipette tip of 2 mm end was filled with mixture paste. For electrical connection copper wire connection was made passed through the edge of the tip.

### 2.5. Voltammetric analysis apparatus

Potentiostat (Metrohm Autolab) PGSTAT 204 was used for voltammetric measurements. All measurements were carried out using a 3 electrodes system; where Ag/AgCl (3 M KCl) was used as reference electrode, platinum (Pt) sheet as counter electrode, and fabricated carbon paste as working electrode.

## 3. Results and discussion

### 3.1. Morphology and structure analysis

**Figure 2:**
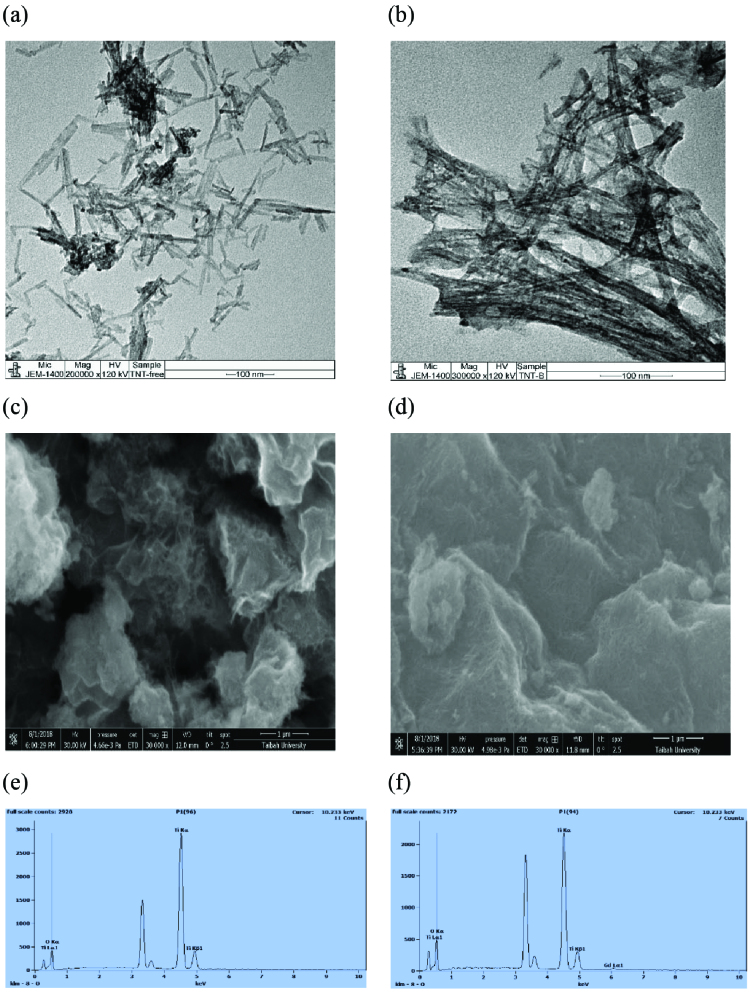
TEM images of (a) undoped TNTs; (b) Gd –TNTs , SEM images of (c) undoped TNTs(d); Gd –TNTs and EDX of undoped TNTs (e); Gd-TNTs (f).

Synthesized TNTs and Gd-TNTs have uniform and hollow multiwall structure, Figures 2a and 2b. The tubular structures of TNT have an outer diameter around 6.5 to 10.6 nm, the length of about 51 nm and Gd-TNTs are in the range 6.1 nm–14.2 nm diameter of range. This is agreed with SEM images in Figures 2c and 2d. where the samples are aggregate as thread network. EDX analysis, Figures 2e and 2f, shows uniform distributions of about 2.92% of Gd in Gd-TNTs.

**Figure 3:**
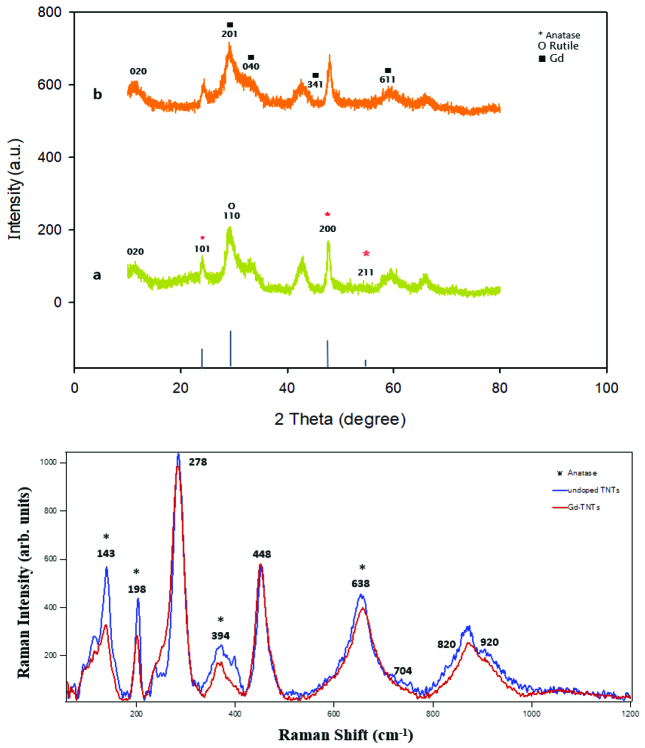
XRD patterns (a) and Raman spectrum (b) of TNT and Gd-TNTs.

The crystal phase of TNTs and Gd-TNTs was identified by XRD. Diffraction peaks of TNT on Figure 3a observed at 24.90°, 48.10°, 55.91°, are diffractions of (1 0 1), (2 0 0) and (2 1 1) crystal planes of anatase TiO2 , respectively (JCPDS, card no.: (00-021-1272). Meanwhile there are characteristic peaks of Gd observed at 29.28°, 31.64°, 47.89°, and 58.72°assigned to (2 0 1), (0 4 0), (3 4 1), and (6 1 1), respectively [35], owes to Gd ion as Gd2 TiO5 (JCPDS, card no.: (00-021-0342).

Figure 3b shows the same strong peaks of Raman spectra obtain of synthesized sensors. The peaks observed at around 143 cm^−1^ (E_1g_) , 198 cm^−1^ (E_2g_), 394 cm^−1^ (B_1g_), and 638 cm^−1^ (E_3g_), respectively, were attributed to anatase TiO_2_ which agree with previous studies [35, 36] and reveal the characteristics of the anatase phase of the sensors. This result is also an evidence to verify that Gd introduced into the lattice or interstitial site of TiO_2_.

The band at 278 cm^−1^ was assigned to the stretching vibration of Ti-O-K bonds and the bands at 198 cm^−1^ and 394 cm^−1^ corresponded to anatase Ti-O-Ti [35,37]. The band at 448 cm^−1^ was related to Ti-O-Ti crystal phonons [38]. The bands at 704 cm^−1^ and 820 cm^−1^ corresponded to covalent Ti-O-H bonds [39], and the band at 920 cm^−1^ was assigned to surface Ti-O-K vibrations [35]

### 3.2. FTIR analysis

**Figure 4:**
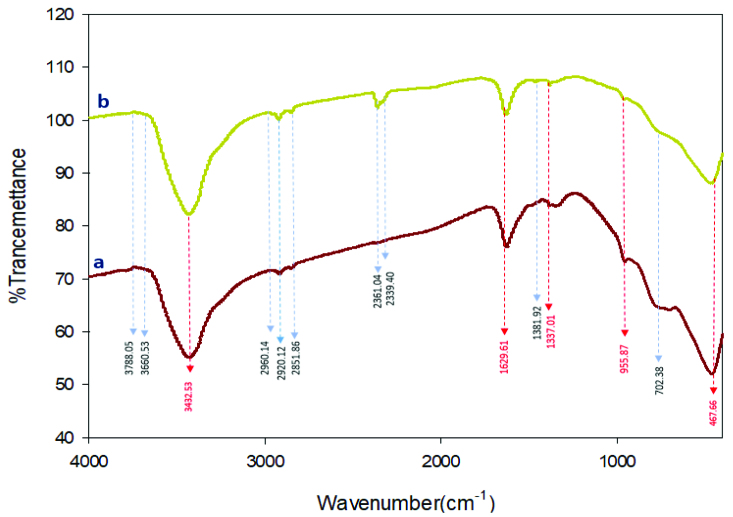
Infrared spectrum of TNTs (a); Gd–TNTs (b).

IR spectra of TNT (a) and Gd-TNT (b) are depicted in Figure 4. Both spectra display the broad band at around at 3432.53 cm^−1^, corresponding to the surface adsorbed water and hydroxyl groups in tubular structure sensors. The large amount of hydroxyl groups on sensors wall enhance their performance for the photo excited electrons capture and profiled the holes to produce the reactive oxygen species in the photocatalytic [40]. The bands at 1627.87 cm^−1^ and 955.87 cm^−1^ can be attributed to the H-O-H bending vibration of the adsorbed water. For synthesized sensor, another typical band at around 446±4 cm^−1^ is originated from the Ti-O-Ti band of TNT and to the asymmetric of Gd-Ti-O in doped Gd-TNT [41].

### 3.3. Electrochemical performance of fabricated electrodes

**Figure 5:**
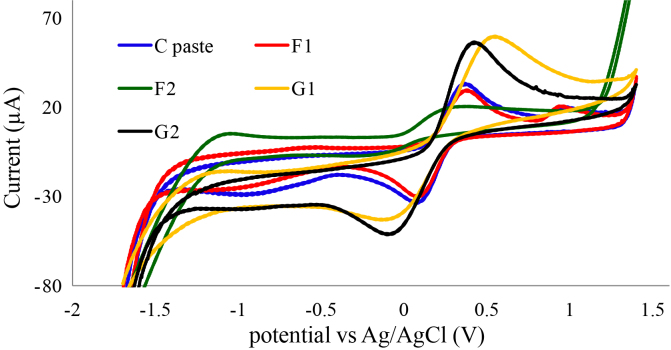
Cyclic voltammograms of 1.0 ×10^−3^ M K_4_ [Fe(CN)_6_], CPE scan rate .0.1V S^−1^.

Cyclic voltammetry was carried out to study the electrochemical performance of fabricated electrodes. Figure 5 shows voltammograms of 1.0 ×10^−3^ M K_4_ [Fe(CN)_6_] with fabricated working electrodes. It could be concluded that doping TiO_2_ with Gd enhances both anodic and cathodic peak currents, where G2 electrode anodic peak current reaches 56 μA compared to 31 μA for bare C paste electrode. Furthermore, G2 cathodic peak current reaches 52 μA which is the highest compared to other studied electrodes. According to the voltammograms of G1 and G2 of Figure 5, it can be concluded that increasing Gd-TNT portion in the fabricated electrodes has a positive impact on electrode sensitivity.

**Figure 6:**
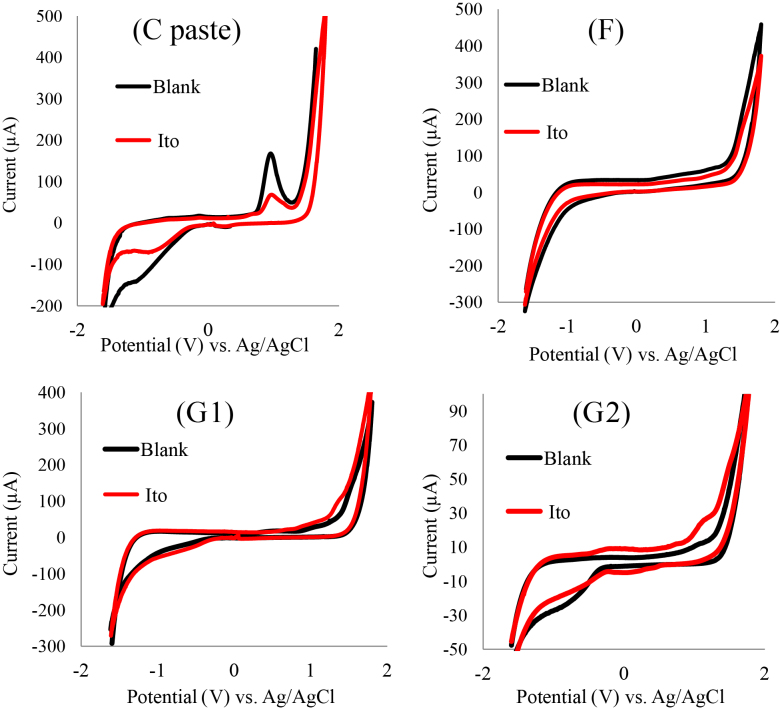
CV Comparison between working electrodes (pure carbon paste, F, G1, and G2) for 0.20 mg/mL itopride assay in 1 M Na_2_SO_4_ supporting electrolyte and 1M Na_2_SO_4_ (blank), scan rate 0.1V S^−1^.

A comparison has been established between fabricated working electrodes for itopride pharmaceutical formulation assay. The voltammograms in Figure 6 show significant difference in performance between studied working electrodes. Figure 6 shows drastic increase in the anodic peak current of G2 working electrode for itopride compared to G1, bare C paste, and (F) electrodes.

When G2 was used as working electrode it showed ΔEp of 1.1 V; where (ΔEp = Epa – Epc) , which is greater than the value of 59/n mV expected for a reversible system [42] suggesting that itopride with G2 working electrode has irreversible behavior in aqueous medium.

### 3.4. Influence of scan rate (υ):

**Figure 7:**
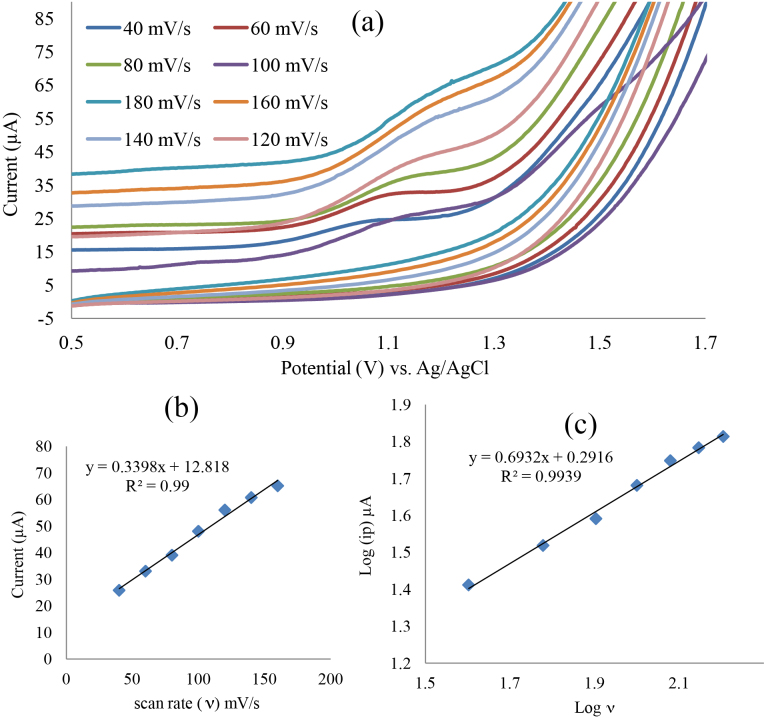
(a) Effect of scan rate on the CVs of 0.20 mg/mL itopride in 1M Na2 SO4 supporting electrolyte, G2 working electrodes, in the range of 40–180 mV/s; (b) Plot of ip vs. ? (c) Plot of log I vs. log ?.

Itopride oxidation mechanism was investigated by study the effect of scan rate on the electrode response. Applied scan rate is ranging from 40 to 180 mV/s. Results are summarized in Figure 7, which indicated that as scan rate increases, anodic peak current increases (Figures 7a and 7b), furthermore it shifts the anodic peak potential (Epa) positively (Figure 7a), which interprets the irreversibility of the electrode process. Figure 7c shows log (ip) vs. log ( υ ) plot which verified linear relationship with slope 0.693, slope value is closer to 0.5, the theoretical value indicated redox process controlled typically by diffusion mass transport only, rather than 1.0 value which typically indicated redox processes controlled by adsorption [42,43].

### 3.5. Analytical performance

**Figure 8:**
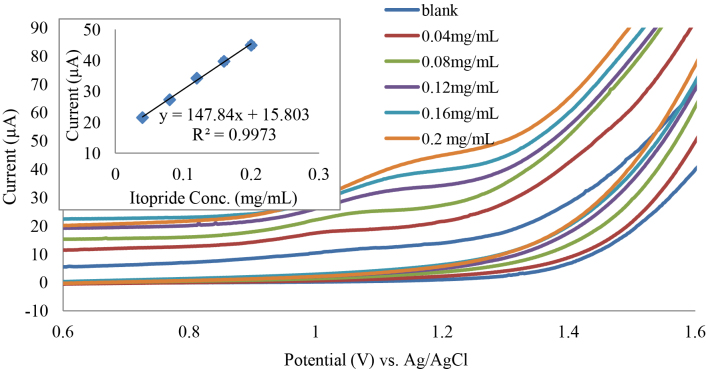
CV study of itopride (0.04–0.20 mg/mL), G2 working electrode, Na_2_SO_4_ (1 M) supporting electrolyte, scan rate 0.1V.s^−1^, each concentration has been done triplicate.

To evaluate the performance of fabricated sensor (G2), a calibration curve was established in the acquired optimum conditions for itopride assay in a pharmaceutical formulation. Figure 8 shows cyclic voltammograms of itopride in a pharmaceutical formulation (0.04–0.2 mg/mL). Standard calibration curve illustrates high correlation (R²= 0.9973) in addition to high sensitivity. Each concentration has been done triplicate with relative standard deviation (RSD) of all concentrations less than 1%. Limit of detection (LOD) and limit of quantitation values (LOQ) found to be 2.9 and 23.0 μg.mL^−1^, respectively. Where LOD and LOQ of itopride were determined based on signal-to-noise ratio of 3 and 10, respectively.

Table 2 shows a comparison between present work and other methods used for itopride determination. This comparison includes precession and LOD. Data in Table 2 indicate that CV analysis of itopride using Gd-TNT electrode has a comparable LOD and precession with chromatographic and spectroscopic methods.

**Table 2:** Comparison in LOD and precession between present study and other methods used for itopride determination.

Method	LOD	Precession	Reference
RP-HPLC fluorescence detection	5 ng/mL	2.81%	9
RP-HPLC/UV detection	12 ng/mL	0.87%	4
Potentiometric	3.98 μM	0.658%	15
UV Spectroscopy	0.5–1.5 μg/mL	0.05%	12
UV–visible spectroscopy	-	1.48%	13
CV-GC electrode	3.50 μg/mL	1.02%	17
CV-C paste Gd-TNT electrode	2.90 μg/mL	0.82%	Present work

## 4. Conclusions

In the present work, TiO_2_ nanotubes have been synthesized and doped with Gd element, and then it has been fully characterized. A composite of carbon paste modified with Gd doped TiO_2_ nanotubes electrode have shown higher sensitivity compared to bare and undoped TiO_2_ nanotubes carbon paste electrode. When Gd doped TiO_2_ nanotubes electrode has been applied for cyclic voltammetry of itopride in a pharmaceutical formulation, it has shown high performance compared to commercially available electrode.
